# Behavioral Health Professionals’ Perceptions on Patient-Controlled Granular Information Sharing (Part 2): Focus Group Study

**DOI:** 10.2196/18792

**Published:** 2022-04-20

**Authors:** Julia Ivanova, Tianyu Tang, Nassim Idouraine, Anita Murcko, Mary Jo Whitfield, Christy Dye, Darwyn Chern, Adela Grando

**Affiliations:** 1 School of Human Evolution and Social Change Arizona State University Tempe, AZ United States; 2 College of Medicine University of Arizona Tucson, AZ United States; 3 College of Health Solutions Biomedical Informatics Arizona State University Scottsdale, AZ United States; 4 Jewish Family and Children's Services Phoenix, AZ United States; 5 Partners in Recovery Phoenix, AZ United States

**Keywords:** behavioral health, patient information, granular information, electronic health record, integrated health care, electronic consent tool

## Abstract

**Background:**

Patient-directed selection and sharing of health information “granules” is known as granular information sharing. In a previous study, patients with behavioral health conditions categorized their own health information into sensitive categories (eg, mental health) and chose the health professionals (eg, pharmacists) who should have access to those records. Little is known about behavioral health professionals’ perspectives of patient-controlled granular information sharing (PC-GIS).

**Objective:**

This study aimed to assess behavioral health professionals’ (1) understanding of and opinions about PC-GIS; (2) accuracy in assessing redacted medical information; (3) reactions to patient rationale for health data categorization, assignment of sensitivity, and sharing choices; and (4) recommendations to improve PC-GIS.

**Methods:**

Four 2-hour focus groups and pre- and postsurveys were conducted at 2 facilities. During the focus groups, outcomes from a previous study on patients’ choices for medical record sharing were discussed. Thematic analysis was applied to focus group transcripts to address study objectives.

**Results:**

A total of 28 health professionals were recruited. Over half (14/25, 56%) were unaware or provided incorrect definitions of granular information sharing. After PC-GIS was explained, all professionals demonstrated understanding of the terminology and process. Most (26/32 codes, 81%) recognized that key medical data had been redacted from the study case. A majority (41/62 codes, 66%) found the patient rationale for categorization and data sharing choices to be unclear. Finally, education and other approaches to inform and engage patients in granular information sharing were recommended.

**Conclusions:**

This study provides detailed insights from behavioral health professionals on granular information sharing. Outcomes will inform the development, deployment, and evaluation of an electronic consent tool for granular health data sharing.

## Introduction

Patient-directed selection and sharing of health information “granules,” that is, the lowest level of health information considered significant (eg, diagnoses, laboratory results, medications), is known as patient-controlled granular information sharing (PC-GIS) [1-6]*.* With the growth of integrated physical and behavioral health care and its reliance on health data sharing, the Office of the National Coordinator for Health Information Technology has promulgated recommendations for PC-GIS by suggesting the implementation of electronic consent tools [5,6]. This model permits the selection and sharing of health information “granules” with patient-specified institutions or personnel for distinct purposes [7] and creates a foundation of trust and transparency among patients, providers, and data stewards [6,8]. While rudimentary ethical guidelines for PC-GIS exist, more comprehensive research is needed [9-11] to harmonize health professionals’ needs with patient choice in an electronically mediated data segmentation environment and, ultimately, a PC-GIS tool.

Literature regarding PC-GIS and granular consent has predominantly focused on the patient perspective [4,7,12]. Outcomes from these studies show that patients view PC-GIS and granular consent positively, especially with respect to sensitive electronic health record (EHR) information [7]. Of the PC-GIS studies, few use actual patient EHR data [7,12-15]. In a 2015 study, Schwartz et al [16] observed how patients enact granular control over their EHR data in a primary care setting. Of the 105 participants given granular sharing control, 45 (43%) chose to limit access to at least one professional, with varying preferences on the type of information restricted. While patients viewed record-sharing control positively (94.3%), they believed that such control may affect their relationship with health professionals (48.6%) [16,17]. Neves et al [15] and Papoutsi et al [18] studied EHR information sharing for research purposes and noted that while patients appear to be concerned about the security of EHR data when shared, health professionals—perhaps incorrectly—worry about patients’ perceived unwillingness to share EHR information.

A 2020 systematic literature review by Soni et al [19] found only 8 peer-reviewed articles on PC-GIS and only 1 that considered the health professional perspective [20]. To understand primary care professionals’ responses and perceptions, Tierney et al [20] supplemented the study by Schwartz et al [16] by reporting professionals’ perceptions and the frequency with which these professionals “broke the glass,” or overrode patients’ sharing preferences, to access additional patient information. The health professionals in this study were based in a general internal medicine clinic—8 physicians, 4 clinical nurse assistants, 3 physician assistants, 2 nurse practitioners, 5 nurses, and 9 medical assistants. The 31 participating health professionals “broke the glass” 102 times, and 90% of these instances were for patients not enrolled in the study [20]. Professionals “broke the glass” for 14% of the total study patients but never “broke the glass” for patients who did not redact information [20]. Of the 24 professionals who responded to their poststudy survey, 63% responded “strongly agree” to the statement “restricting access to all or part of a patient’s EHR will likely reduce the quality of care I deliver” while agreeing that patients should have such control [20]. Although Schwartz et al [16] and Tierney et al [20] provided patient and health professionals’ survey responses, they did not provide patient rationale and included minimal provider rationale for the PC-GIS choices.

Prior literature shows that patients may restrict access to potentially sensitive health data because of stigma or fear of discrimination [21-23]. The 2020 research by Soni et al [19,24] reported a mixed-method approach using patients’ own EHR information to assess preferences for PC-GIS in behavioral health care settings. The study outlined a card-sorting, semi-structured interview methodology of asking 25 English- and Spanish-speaking patients who were diagnosed as having general behavioral health (GBH) disorder and serious mental illness (SMI) from 2 integrated care clinics to categorize 30 items from their own EHRs as sensitive or nonsensitive. Nonsensitive data included all general physical health items, while the sensitive data groups were based on the Substance Abuse and Mental Health Services Administration sensitivity groups: alcohol use and alcoholism, communicable diseases, drug abuse, genetic information, mental health, other addictions, and sexual and reproductive health [19,24,25]. Participants were asked to classify the 30 items by sensitivity, that is, not sensitive, somewhat sensitive, sensitive, and then to exercise PC-GIS. Participants considered mental health (76%), sexual and reproductive health (75%), and alcohol use (50%) as sensitive categories. They were willing to share items related to other addictions (100%), genetic data (95.8%), and nonsensitive information (90.5%) [19]. Sharing preferences and sensitivity classifications did not significantly correlate [19]. Further, the study showed that participants’ understanding and views on sensitivity, categorization, and sharing of information were diverse and that such diversity could impact use of an electronic consent tool [24]. Participants’ personal circumstances impacted their sensitivity classifications and sharing preferences, but classification and sharing were approached independently [19,24].

Participants from the study conducted by Soni et al [19,24] and Schwartz et al [16] chose to share their data with all (48%) or some health professionals (52%). Health professionals included primary care providers, specialty care physicians, pharmacists, nurses, case managers, counselors, and medical assistants. The researchers suggested that a study focusing on professionals’ perceptions of PC-GIS would enrich their findings and further inform understanding of the elements needed to support PC-GIS.

It is critical to understand professionals’ perspectives to develop granular consent systems that balance patient desires with the information needs of health professionals. This study focuses on health professionals employed by the integrated care clinics used by the study conducted by Soni et al [19,24] and uses the study’s results in the focus group design [20].

Our study used qualitative data analysis to gain insight into health professionals’ perspectives of PC-GIS, specifically the (1) understanding of and opinions about PC-GIS; (2) accuracy in assessing patient-directed redaction of medical information; (3) reactions to patient rationale for health data categorization, assignment of sensitivity, and sharing choices; and (4) recommendations to improve PC-GIS.

Results and recommendations from our study and Soni et al’s research [19,24] will inform the development of a PC-GIS tool, My Data Choices*,* inspired by similar technology developed by the Substance Abuse and Mental Health Services Administration [25]. My Data Choices will be pilot tested at the same integrated care settings at which this study was conducted.

## Methods

### Clinical Settings

Study data were collected at 2 integrated care clinics [19,24]. One facility focuses on caring for patients diagnosed as having GBH disorder, and the other facility specializes in caring for those diagnosed as having SMIs. These facilities will be referred to as GBH Facility and SMI Facility throughout the manuscript. The GBH Facility staff had 85% nonprescribers and 15% prescribers, while the SMI Facility staff had 90% nonprescribers and 10% prescribers. Prescriber designation is determined by the Secretary of Labor definition [26,27]. Participants were selected to achieve representative samples at each facility.

### Participants

The study was approved by the Arizona State University Institutional Review Board (#00010309). Participants who spoke English, were 21 years or older, worked closely with patients (primary care providers, psychiatrists, nurses, case managers, etc), and are currently or were recently involved in the previous year in consent processes at the 2 facilities were included. The SMI Facility participants were self-selected through a flyer distributed by a facility representative. A representative sample of professionals was solicited at each facility for each focus group. All participants received a $75 gift card as compensation on completing at least an hour and a half of the 2-hour focus group.

### Focus Groups

**Figure 1:**
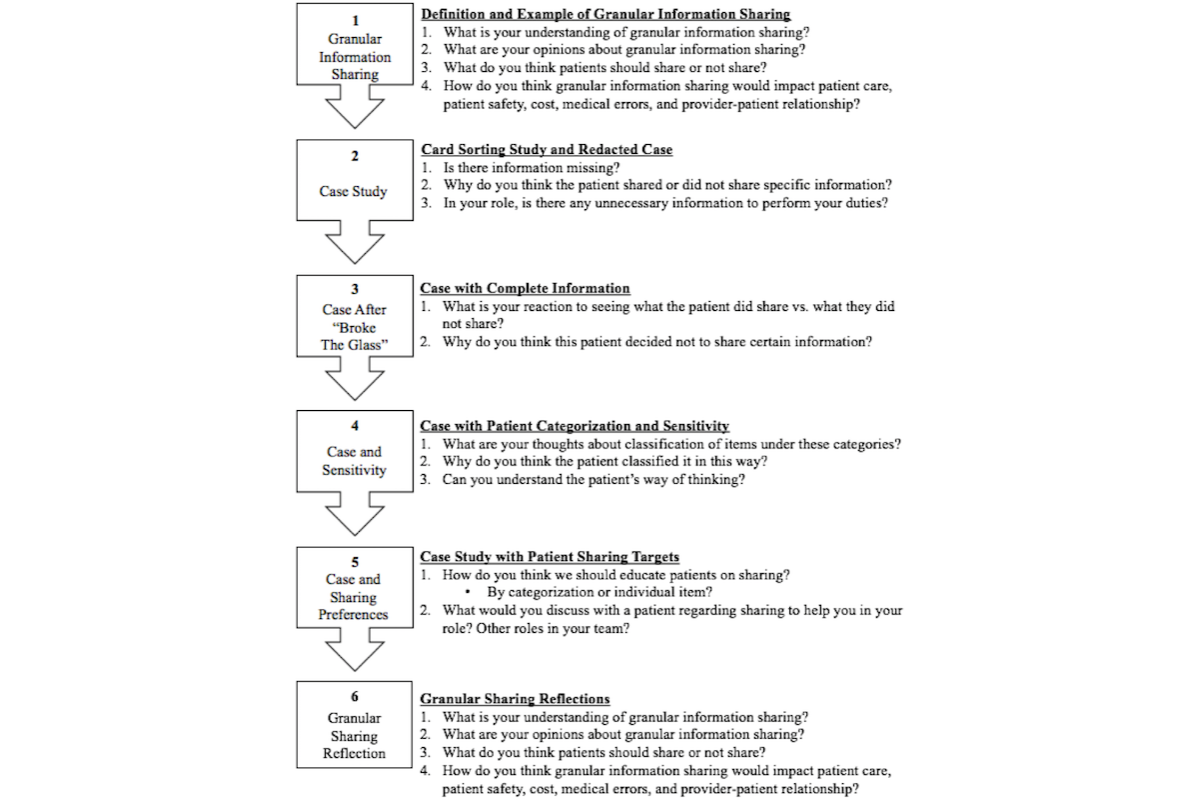
Focus group flow by section (1-6) with corresponding target concepts for each section.

**Figure 2:**
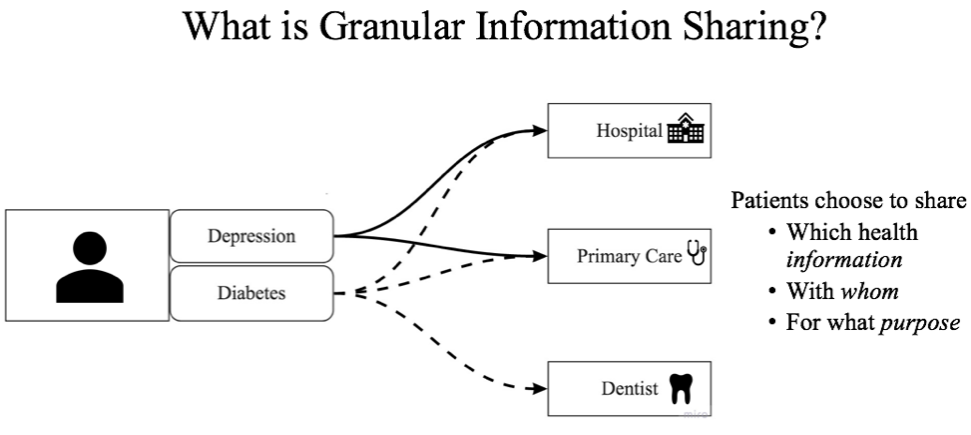
Example used to explain patient-controlled granular information sharing.

Four 2-hour focus groups were conducted and audio recorded at each facility. Each focus group comprised 7 health professionals. Pre- and postsurveys, adapted from Tierney et al [20], were administered. The 6-section format of the focus groups and corresponding questions are illustrated in Figure 1. Content included PC-GIS didactics and an actual patient case from the Soni et al study [19,24] (Figure 2). In the example case, the patient chose to share information related to depression and diabetes with hospital physicians and the primary care doctor but shared only diabetes information with their dentist. The first author led the focus groups, and 2 observing researchers documented visual and verbal information to ensure all information from focus groups was captured in the final analysis.

#### Section 1

Baseline comprehension of granular information sharing was assessed at the start of the focus group (question 1). “Granules” was later defined as “the lowest level of health information considered significant, such as diagnoses, laboratory results, or medications” [1-6] and granular information sharing occurs “when a patient identifies specific health information (granules) to share with or withhold from, specific professionals, entities or organizations, and directs how that information will be used” [2-6,8,27]. Figure 2 was discussed, followed by questions 2-4.

#### Section 2

**Figure 3:**
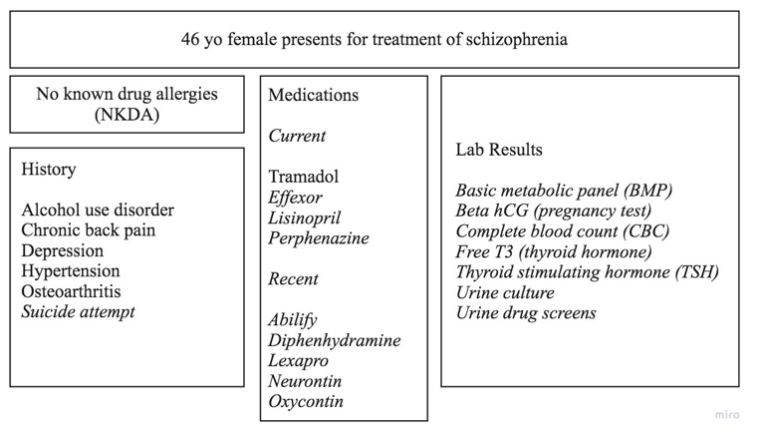
Case study from Soni et al [19,24] presented with PC-GIS redaction (Section 2) followed by “breaking the glass” (Section 3), which is simulated by revealing the previously hidden items denoted by italics. PC-GIS: patient-controlled granular information sharing; hCG: human chorionic gonadotropin; T3: triiodothyronine; yo: year-old.

The methods and results from the Soni et al study [19,24] were used to demonstrate how actual patients exercised granular information sharing, and a patient-redacted case was presented without disclosing that redaction had occurred (Figure 3). A singular case study was chosen to allow participants enough time to evaluate all aspects of PC-GIS. The specific case study was chosen as an example of how patients who did choose to restrict some information utilized PC-GIS in the Soni et al study [19,24].

#### Section 3

The patient case was presented again, this time with the previously redacted health data items [24], and possible patient motivation for withholding this data was discussed (Figure 3).

#### Section 4

**Figure 4:**
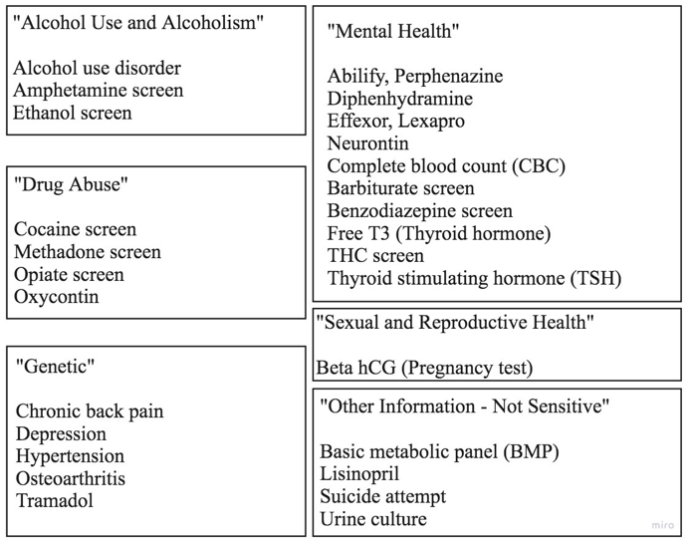
Classification of data as sensitive and categorization of health information by patients from the Soni et al case study [19,24] presented after the redaction is revealed (Sections 4 and 5). hCG: human chorionic gonadotropin; THC: tetrahydrocannabinol; T3: triiodothyronine.

Data categorization and sensitivity classifications by patients were shared [19,24]. The discussion centered on patient rationale for the presented choices (Figure 4).

#### Section 5

The actual published patient rationale for sharing or withholding specific health data items was presented and discussed. Additional details from the paper on data categorizations, sensitivity classifications, and sharing preferences were provided if requested [19,24].

#### Section 6

After they reflected on the prior sections and the hypothetical tool presented, participants were asked to share opinions and to make recommendations to those seeking to implement PC-GIS.

### Analysis

Focus group audio recordings were transcribed using Transcribe (Wreally LLC) and then sequentially screened by 3 researchers for accuracy. Notes from onsite researchers, that is, notes on nodding and facial expressions, were added to the transcripts. Transcriptions were analyzed using Braun and Clarke’s thematic analysis guidelines and anthropological methodology [28,29]. Six iterations of digitally assisted coding (MAXQDA, VERBI GmbH) were performed. The unit of analysis was an individual participant’s statement in paragraph form, and themes were identified through repetition and frequency. One researcher coded for and defined emerging themes into a codebook. The output was iteratively revised by 3 researchers and organized according to the focus group segments, as represented in Figure 1. Although the focus group leader prevented dominance by a single participant, transcript codings were calculated per participant to determine whether egalitarian engagement was maintained. Each focus group participant was coded as a separate entity, thus any codes attributed to them could be measured. At conclusion of coding, participants’ actual attributed codes were juxtaposed to the expected number of codes per person for each focus group. Coding assessments provided qualitative and quantitative insight into participant rationale for PC-GIS opinions with outcomes. Pre- and postsurvey analysis is described in part 1 of this study [30].

## Results

### Demographics

The participant group included 23/28 (82%) nonprescribers and 5/28 (18%) prescribers (physicians and nurse practitioners) based on prescriber criteria (Table 1) [26,27]. All participants (28/28, 100%) completed the presurvey and 27/28 (96%) completed the postsurvey (1 participant was on call and departed prior to focus group conclusion).

While the focus groups each had 1 participant who engaged more than anyone else (outside the group average), these participants were individuals who had administration or managerial duties and helped facilitate the discussion of the rest of the group.

**Table 1:** Health professionals’ roles and representation (N=28).

Role type		Professionals, n	GBH^a^ facility, n	SMI^b^ facility, n
**Nonprescribers**				
	Counselors	4	3	2
	Nurses	3	1	2
	Rehabilitation specialist	3	1	2
	Case managers	3	1	2
	Clinical coordinators	3	3	0
	Administrators	3	0	3
	Peer mentors	1	0	1
	Medical assistants	1	0	1
	Discharge planners	1	0	1
	Social workers	1	1	0
**Prescribers**				
	Physicians	3	2	1
	Nurse practitioners	2	2	0

^a^GBH: general behavioral health.

^b^SMI: serious mental illness.

### Health Professionals’ Understanding and Opinions of Granular Information and Sharing

Thematic analysis coding of individual participant contributions demonstrated overall absence of single participant dominance regarding codes. Thematic analysis results of health professionals’ knowledge and perceptions of PC-GIS before (section 1) and after (section 6) the focus group were compared. Prompts from these sections were assessed for comprehension of the granular information concept (unaware of, correct, or incorrect definition) and for opinions (positive, negative, mixed, or not applicable) on how PC-GIS could impact care delivery.

Baseline comprehension of PC-GIS yielded 25 relevant codings in section 1 (Multimedia Appendix 1). Just over half (14/25, 56%) of the participants were unaware of or provided incorrect definitions of granular information sharing: “I don’t have any understanding of it.” After a brief explanation (Figure 2), participants demonstrated 100% (15 codings) comprehension of PC-GIS. By conclusion of the focus groups (section 6), all participants demonstrated excellent comprehension of PC-GIS (100%, 8 codings) with nuanced discussion (35 codings; Multimedia Appendix 1) of concerned, positive, and mixed opinions.

**Figure 5:**
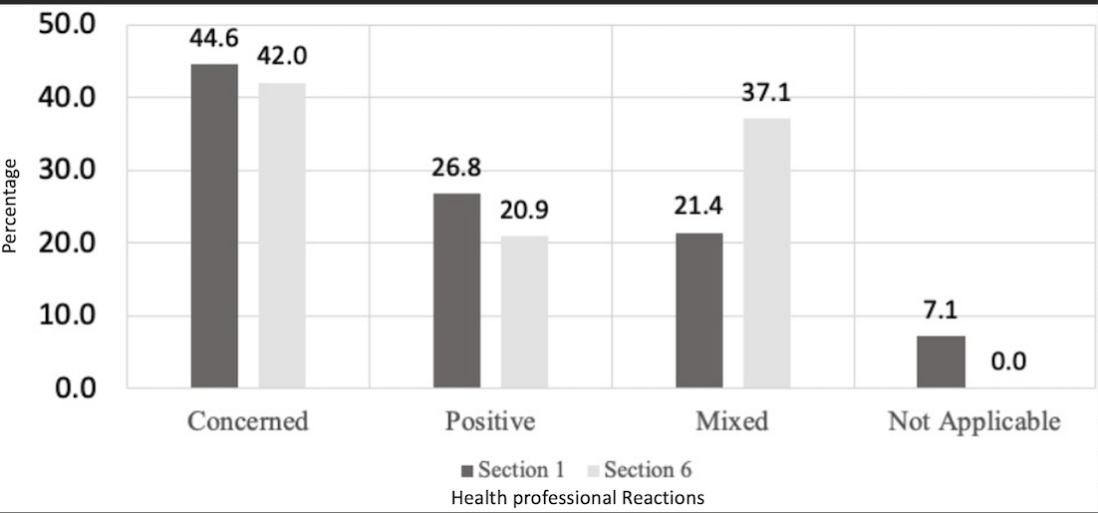
Initial and concluding reactions from the focus groups to patient-controlled granular information sharing (Sections 1 and 6, compared). Numbers signify percentage per category. Rounded data do not always add up to 100.

There was a visible change from the initial to the concluding reactions to PC-GIS within focus groups with respect to mixed opinion (Figure 5). For the “not applicable” codings from section 1, participants expressed neither a definition nor an opinion. They did discuss foreign language and education of patients as issues in granular information sharing. Positive and mixed outlooks focused on patients’ rights and choices, alleviation of stigma, rationalization of patients’ choices, and streamlining of communication. When asked to imagine PC-GIS from the patient perspective, most participants (20/22, 91% of instances attributed to positive codings) expressed a more supportive view of granular sharing.

Patient safety and the ability to provide successful treatment were frequently cited (55/99, 56% of codings from sections 1 and 6) in the discussion about patient-directed redaction:

It can be life threatening. There are some medications that can be prescribed that if you combine like an MAO inhibitor with a pain medication, it can be fatal…you [patient] may not think it’s important, but you’re not really trained, you don’t have the background to know what’s important per se or what isn’t. It is difficult to help your patient understand that, a lot of education. (Agreement throughout).

Concerns about patients’ rights and patients’ perspectives were raised in both sections 1 and 6, as were complexities of implementing a PC-GIS tool that adequately balanced patients’ rights with health professionals’ needs. The transition in perception from sections 1 to 6 was striking, with an increase in the proportion of participants with mixed opinions.

I actually have a different opinion [than] when I started. I mean, it depends on what angle you’re coming at. It really does because if you’re a patient, I get it. I get it why you don’t want to share everything. But from a provider nurse standpoint, safety becomes a factor. And that’s when more information should be shared as much as possible.

Another participant noted that a granular sharing tool may help reduce the cost of care, if appropriate sharing was enacted. While participants with concerned opinions prioritized health professionals’ perspectives, participants with mixed opinions weighed patient and health professionals’ perspectives equally.

### Health Professionals’ Assessments of Patient Health Information Redaction

Section 2 focused on health professionals’ recognition of record redaction (32 codings; Multimedia Appendix 1). Most participants (26/32, 81%) accurately identified that some information was missing from the presented health record. Still, 19% (6/32) of codings showed that participants were unaware (3/32, 9%) of the redaction or were uncertain (3/32, 9%) if the information was complete; they explained that they are accustomed to working with incomplete or absent patient information.

All participants responded with varying concern about missing information (Table 2). They identified the data categories that were redacted or incomplete and, in actual practice, would discuss suspected redaction or withholding of information with the patient (51 total codings). Specifically, the patients’ discussion (27 codings) with the participants (6/27, 22% of patient discussions) would include rationale for the information: “Tell them [patients] the reason why we’re asking, the importance of it, and to help them understand why we need the information.” Others (3/27, 13% of patient discussions) included the suggestion that use of standard facility procedures (eg, intake questionnaires) could aid in finding the needed information: “…As a case manager, we do a comprehensive assessment...hey, you don’t have to be honest but at least I asked.”

**Table 2:** Health professionals’ reactions after redacted material was revealed (N=51; section 2); rounded data do not always add up to 100.

Code	Coding, n (%)	Exemplar quotes
Conducted patient discussion	27 (52)	“I would just be honest and say, so I see that you have a diagnosis here that you’re presenting for treatment of schizophrenia but I don’t see that you’re currently prescribed an anti-psychotic. Are you currently taking one? Have you taken one in the past? Did we possibly forget to list any current medications that you may have forgotten? I would just if it were me, address it pretty upfront.” [nonprescriber]
Expressed concern	18 (35)	“Because if she’s using, yeah, if she’s using both then that could be potentially deadly.” [prescriber]
Expressed need for information	6 (11)	“But yeah, there’s a lot of information missing. I would want a more complete social, family history, hospitalization history, past medications. It’s again, we don’t know what’s worked, what hasn’t worked and we’re just kind of now starting from scratch again…if that’s all that’s there, it’s not enough to move forward with treatment without more information.” [prescriber]

### Health Professionals’ Perceptions of Patient Rationale for Sharing Decisions

In sections 3 and 4, we assessed participants’ reactions to patient sharing rationale. Participants (48 codings; Multimedia Appendix 1) reacted to the redacted content revelation by expressing the need to know this information (33/48, 68%), surprise (9/48, 18%), and no surprise (6/48, 12%). While many participants noted that they would question patients or use alternate sources to gain need-to-know information, they were skeptical that all the information necessary for optimal and safe treatment of this patient had been identified: “As the therapists doing an assessment, we’re not necessarily going to even get to a question that hits on all of the panels that are missing.” Similarly, participants (3/9, 33% of those surprised) also noted that the data categorizations chosen by the patient “did not make sense.” They were also surprised by how the patient applied these categories to make sharing decisions.

When participants were asked to postulate the patient sharing rationale (Table 3), the results (30 codings) coalesced around stigma and fears, purposeful omission, consideration of data to be irrelevant, lack of clarity on the information that needs to be shared, and symptoms. We then asked the participants to react to the data sensitivity classification assigned by patients (section 4). Participants registered 3 main types of reactions (62 codings): did not understand (41/62, 66%), considered patient incorrect (15/62, 24%), and considered patient correct (6/62, 9%). Of note, all participants who reacted positively to some patient classifications did not agree with the patient’s sharing decisions and found the documented patient rationale helpful in understanding the patient’s decisions.

Patient data self-categorization using the 6 data categories (77 total codings; Multimedia Appendix 1) resulted in genetic (17/77, 22%), mental health (14/77, 18%), drug use (9/77, 11%), alcohol use (9/77, 11%), sexual and reproductive health (3/77, 3%), and other information (25/77, 32%). Participants focused on the category “other information” that included the topic of attempted suicide (22/25, 88% of all “other information” discussions). Participants found the actual patient explanation for classifying the suicide attempt item into “other information” rather than “mental health” to be particularly relevant: “took a whole bunch of pills” [19]. Participants considered that the patient thinks “they’re fixed” (nonprescriber) or “they don’t have a good understanding of what mental health is” (nonprescriber). Participants were provided detailed patient rationale as requested, including explanations from other patients in the Soni et al study [24].

**Table 3:** Health professionals’ rationale of the patient’s decision to redact (N=30; section 3); rounded data do not always add up to 100.

Code	Coding, n (%)	Exemplar quotes
Stigma and fear	13 (43)	“I don’t know the culture of this client, but culturally they might be thinking like, “This person thinks I'm crazy or people will think I’m crazy because I take medication so I’m just not going to say anything.” Particularly if it’s a court-ordered client, they may be sharing less because they just want to get their mandates over with and get out of services. And the more they share could keep them wrapped up in services for longer than they want.” [nonprescriber; nonprescribers nod in agreement]
Purposeful omission	7 (23)	“Well, I’m just saying in general, if I go to the PCP, I’m going for one thing, I don’t need 50 other things added on to what I came here for. So, maybe they’re just shutting it down. And like, look, this is what I’m here for and this is what I'm giving you.” [nonprescriber]
Patient considered data irrelevant	5 (16)	“Or is it with the one-time [suicide] attempt, it really didn’t mean nothing. I didn’t really want to do it, so I’m okay now. So, it’s not important to me. It’s not relevant to them.” [nonprescriber]
Patient lacks clarity on the information that needs to be shared	3 (10)	“Like six months into treatment, they suddenly randomly talk about a shopping addiction or something like that that they just never mentioned. And so, I’m sure there’s some things that they don’t realize are important to share [with us].” [nonprescriber]
Symptoms	2 (6)	“There’s the possibility that they’re not 100% compliant with their medication because again, there’s a lot of side effects from medications. And I’m not seeing side effects of medication being prescribed and then there’s the drug screen, so we don’t know how much the person’s self-medicating and taking their meds. So, they may be more symptomatic hence could be more paranoid about sharing the information. So, I’d want to rule that out as well. How symptomatic are they at that particular moment, you know?” [nonprescriber]

### Health Professionals’ Recommendations to Improve PC-GIS

Participants reacted to the description of a hypothetical PC-GIS tool based on the patient case in section 5 (Table 4). They discussed how a patient should be advised to share certain information (39 total codings). Most participants indicated that ensuring transparency, promoting trust, and fostering understanding are key factors for successful PC-GIS. This might be enhanced by periodical review of sharing decisions in a meeting with adequate time for questions and discussion with the provider.

Participants also addressed the need to simplify education material (4/39, 10%), create role-specific information (3/39, 7%), and use examples (2/39, 5%; Multimedia Appendix 1). Behavioral health professionals stressed that additional resources or a different approach may be needed to elicit informed consent for record sharing from a competent patient with active psychiatric symptoms. Participants also acknowledged that their differing roles and professional preparation necessitate the use of targeted materials to support specific sharing discussions. Finally, they underscored the importance of motivating patients to engage in the data sharing process and to understand its impact on safety.

**Table 4:** Health professionals’ recommendations to improve granular information sharing (N=39; section 5); rounded data do not always add up to 100.

Code	Coding, n (%)	Exemplar quotes
Promote trust and understanding	23 (60)	“Absolutely interesting because again, the client isn’t sharing information about their mental health with the people who are designated to help them with their mental health. So again, if that’s the theme then trying to (A) understand what is the motivation for that and (B) is there something that can be done to assist with building some trust? If that’s in some way, you know, if they don’t trust the system or whatever it may be or they’re symptomatic, how can we kind of overcome that barrier in order to get that client's unique needs met?” [nonprescriber]
Other	7 (17)	“I would use a similar grid like that grading, because at a glance, you could introduce something every three months, any updates. Are you still sharing with your pharmacist? Are you still sharing with your own specialty care providers, etc.? Have you mentioned that you have an upcoming appointment with PCP? And a bit something of an alert, definitely, you need to work with the team and send an email.” [nonprescriber]
Simplify education material	4 (10)	“Even having it written down, sometimes it might be too much for somebody who’s having schizophrenia. If I’m hearing voices, I don’t have the patience to sit down either listen or read something. I just want to get it done as soon as possible.” [nonprescriber]
Provide role-specific information	3 (7)	“We also take time to educate because if we have to educate them on everything, there’s thousands of topics to discuss, and we can’t educate or try to educate on things that we’re not competent in. So, I can’t talk to them about medications. I won’t [non-prescriber] because I can’t. It’s not ethical, and it’s not a smart decision. So, you know, if they want the education, then they have to go see their doctor or their nurse practitioner, you know? And then it’s just more steps. But if they’re willing to do it, that’s great. But they have to be motivated to do that.” [nonprescriber]
Provide examples	2 (5)	“Give an example. Because someone with schizophrenia is not going to have the patience to sit there and listen to what each definition is and where it goes.” [non-prescriber; agreement between nonprescribers and prescribers]

## Discussion

### Main Findings

Our analysis of behavioral health professionals’ perceptions of PC-GIS between the start and end of the focus groups demonstrates a shift to mixed opinions from a position of less support (12/56, 21% to 13/35, 37%). Although the terminology and processes of PC-GIS were new to many professionals (14/25, 56%), all participants understood the concept, benefits, and risks associated with PC-GIS after a brief explanation. Additionally, the professionals correctly identified (26/32, 81%) that information was missing (patient-redacted) from the case presentation, with a majority (33/48, 68%) noting that the missing information was necessary for the successful care of the patient. Professionals were perplexed about many patient categorization and sharing decisions (41/62, 66%) and often expressed surprise when the patient rationale for withholding information was shared. Participants’ concerns led to a general commitment to improve the consent process with specific recommendations.

The literature suggests that adoption of EHR and health information exchange has accelerated the importance of consent technology. Emanuel and Emanuel [31] noted that patients and professionals, the key stakeholders, must be part of any process change involving the fine balance between care delivery and individual rights. Trust and transparency are key factors in this delicate relationship [14,27,32-36]. Our results support and expand these findings; namely, the health professionals have defined challenges in PC-GIS implementation (28/35, 79% of final concerned and mixed opinions), attempted to understand patient motivation for redaction, aimed for balance between patient and professionals’ needs, and underscored the need to access necessary health information for successful care delivery (23/39, 60% of all recommendations). The pre- and postsurvey analysis (described separately) [28] shows significant changes (*P*<.05) in health professionals’ opinions toward concern after the focus group [30]. Our qualitative analysis mirrors and provides insight into the increase of mixed opinions with comprehension of PC-GIS as well as recommendations to mitigate mutual concerns related to PC-GIS and avoid friction in the patient-professional relationship.

While there is research on health professionals’ understanding of granular information control, there are no studies measuring baseline PC-GIS knowledge or the effectiveness of an intervention to enhance that knowledge [20,27,35,37]. In our study, behavioral health professionals (14/25, 56%) were either unaware of or provided incorrect definitions of PC-GIS. A brief explanation and example (Figure 2) resulted in 100% (15/15) comprehension of the terms and process. Implementation of PC-GIS requires education of health professionals, and we demonstrated that this can be accomplished with a brief explanation.

Prior literature has focused on whether and how professionals should be notified about patient redactions [9]. The results of the study by Tierney et al [16,20] were inconclusive on provider awareness of redacted information but showed that professionals did not “break the glass” for any of the patients who chose to share all information. In our study, most participants (26/32, 81%) correctly recognized the absence of some data during the case exercise. Therefore, our results suggest that most health professionals may independently surmise that the available data are incomplete; they mentioned that they routinely evaluate patients with incomplete or fragmented records. When redaction or withholding of information is suspected, the health professionals agreed that such information gaps should prompt timely, directed patient discussion. They further noted that patients place themselves at risk for suboptimal treatment and even injury when health data are incomplete at the point of care.

We asked participants to consider information sharing from the patient perspective. Our study compared the health professionals’ postulated rationales with those provided by patients in the Soni et al study [24]. Of the 32 sensitive-information codings from Soni et al [24], half of the patients (50%) said that stigma and fear of discrimination were the reasons for their classifications [19,24]. Our participants agreed that stigma and fear (13/30, 43%) were driving forces for patient data redaction. Soni et al [24] reported that some patients conflate sharing categories with sensitivity by classifying and sharing based not only on comprehension but also on perceived applicability to their own health history. In our study, participants’ insights mirrored patient justifications for purposeful omissions (7/30, 23%), omissions for irrelevancy (5/30, 17%), and omissions because of a lack of clarity on the information that needs to be shared (3/30, 10%). Further, participants did not understand (41/62, 66%) the rationale used by the patient to classify and make sharing choices. When provided with patient rationale from the Soni et al study, the health professionals suggested specific techniques for interacting with patients who had unclear or missing health information. Overall, the health professionals were concerned about patients categorizing and classifying information in a manner incongruent to bioscience and health professionals’ reasoning.

Recommendations for designing PC-GIS tool features comprised the final segment of the focus groups. Participants emphasized that targeted PC-GIS education for behavioral health and integrated care settings must be appropriate for individuals with lower health literacy (4/39, 10%), permit adaption by profession (3/39, 8%), and contain pertinent, patient-friendly examples (2/39, 5%).

Also important for PC-GIS tool design is consideration of patient and provider time constraints, including the resources needed to engage in face-to-face communication. Most PC-GIS studies employed a “break the glass” option for health professionals to access needed information [16,20]. Our participants expressed the need for such a feature. They also suggested periodic meetings with a trusted care professional and creation of an electronic algorithm within the PC-GIS tool to help patients classify and select information for sharing. Feedback from frontline professionals is integral to the development of a digital tool for informed PC-GIS.

### Limitations

The study included a limited number of health professionals, particularly prescribers. Therefore, we cannot comment on differences between prescribers and nonprescribers. However, participant composition in this study reflected the facilities’ overall prescriber-nonprescriber ratios and was representative of role demographics for the respective professional population. The resulting demographics of the focus groups for the facilities showed a diverse representation of the entire care team that a patient relies on within integrated care. Focus groups comprised individuals who were actively engaged in the care delivery team. We recommend that future studies consider the prescriber and nonprescriber aspects that may impact the success of a PC-GIS tool.

While this study used a single representative patient case, sharing of the overall results of the Soni et al study [19,24] provided additional context and insight into how other patients with GBH disorder and SMI categorized, classified, and rationalized their decisions.

Focus groups may include participants that tend to dominate a discussion, especially in the context of existing workplace hierarchy. Efforts were made to avoid this situation. Moreover, focus group codes were evaluated per participant and demonstrated no such effects to significantly skew discussions.

Health professional roles (eg, prescriber, nonprescriber) and patient population (eg, GBH, SMI) may impact the interpretation of and opinions about granular information sharing, its potential impact on care, and how best to provide informed consent [27]. Published literature supports the rapidly evolving trend toward integrated care coupled with the need to improve digital sharing processes [38]. This study provides context and recommendations to help achieve this goal.

### Future Research

Our results are being incorporated in the design and deployment of a PC-GIS tool, My Data Choices. The participants’ recommendations are also being used to develop patient education for the My Data Choices pilot to be launched in several integrated care clinics. The My Data Choices study team is also investigating the impact of trust on PC-GIS.

### Conclusions

This study provides detailed insights from behavioral health professionals about granular information sharing, explores scenarios where patients exercise granular consent choices, and includes suggestions to improve patient education and the consent sharing process. The case-based learning intervention during the focus group improved provider comprehension of PC-GIS terminology and process. Health professionals accurately identified the presence of patient-redacted information gaps and provided concrete recommendations to help patients appreciate the risks and benefits associated with PC-GIS. Outcomes of this study are guiding the development, deployment, and evaluation of an electronic granular consent tool.

## Appendix

Multimedia Appendix 1 Codes from specific sections of the focus groups by code, number of codes, and percentage of codes per section, and example quotes from participants.

## Abbreviations

EHR: electronic health record
GBH: general behavioral health
PC-GIS: patient-controlled granular information sharing
SMI: serious mental illness
