# Supplementary material for: Behavioral Health Professionals’ Perceptions on Patient-Controlled Granular Information Sharing (Part 2): Focus Group Study
Source: JMIR Ment Health. 2022 Apr 20;9(4):e18792. doi: 10.2196/18792 (PMC9069296; doi:10.2196/18792)
Supplement: Multimedia Appendix 1 [file mental_v9i4e18792_app1.docx]

| Codes | Codings (#) | Percentage | Exemplar Quotes |
| --- | --- | --- | --- |
|  |  |  |  |
| Understanding of PC-GIS Before Focus Group (Section 1) | | | |
| Unaware of Definition | 11 | 44 | I don’t have any understanding of it. (non-prescriber) |
| Correct Definition | 11 | 44 | I’m thinking more like bits and pieces you choose, which pieces of information is shared, which is not. (non-prescriber) |
| Incorrect Definition | 3 | 12 | Grand is what I’m thinking, lots of information all around is what I’m thinking. (non-prescriber) |
| Understanding of PC-GIS After Explanation (Section 1) | | | |
| Confirmation of Understanding | 15 | 100 | Okay, I had it backwards. It's about them sharing, deciding what to share with us. (non-prescriber) |
| Reactions to PC-GIS Explanation (Section 1) | | | |
| Concerned | 25 | 44.6 | It’s just someone in the SMI population…might not have the capacity to understand how dangerous it is. I think maybe that prescribers should be automatically given that information with medication that they need to treat someone. (non-prescriber) |
| Positive | 15 | 26.8 | I think it's a good thing because I know if I had depression and there's was no good reason for my dentist to know that…it's my choice I guess and that's what's nice. (non-prescriber) |
| Mixed | 12 | 21.4 | I could see from the patient side being able to decide bits and pieces of what goes out…depending on where it goes, the stigma follows, especially with mental health, the stigma follows. Yet, from working in that field, I could also see the danger part that you guys were talking about and the safety and so forth. I don’t – not everyone needs to know about my mental health diagnosis, but they kind of do. So, I don’t know. I guess I just kind of ended up in the middle. (non-prescriber) |
| Not Applicable | 4 | 7.1 | I also think there is a language barrier about sharing and consenting because we have a lot of clients who are Arabic speaking and it's like doing intakes with the phone interpreter...They don't seem to understand. (non-prescriber) |
| *Reconfirmation* of PC-GIS Understanding (Section 6) | | | |
| Confirmation of Understanding | 8 | 100 | Being able to select what information you want to share with which provider. (prescriber) |
| Reactions to PC-GIS at Focus Group Conclusion (Section 6) | | | |
| Concerned | 15 | 42 | Yeah, I would be worried that the patient doesn't share the right information with the right provider. (prescriber) |
| Mixed | 13 | 37.1 | It’s health information considered significant. Well, who's considering its significance, the client or the professional? That might've been one reason why a lot of information wasn't shared is that that wasn't significant to the client at the time of the intake. So, I still think that it's very complicated and complex. (prescriber) |
| Positive | 7 | 20.9 | Well, right now there’s nothing, so something is better than nothing. Even if a patient will be willing to share a minimal amount of information that’s better than what we have now. I would just be grateful for anything really, I mean, if that’s all the patient wants to share, and that’s more than you’d have otherwise. (non-prescriber) |
| Assessment of Health Information Redaction (Section 2) | | | |
| Missing Information | 26 | 81.3 | We go by treatment plan. I mean it's a lot of assessments. I mean it's a lot of things is missing in this chart for us to assess the individual to get to meet their needs. (non-prescriber) |
| No Missing Information | 3 | 9.4 | I agree that this looks like it's not a complete chart for me. But I guess it could also be possible too, maybe you have a client who's not really involved in like the PCP side and they have these diagnoses, but they're not taking any medication and not following up with that PCP to treat those especially since we know our SMI population is not really diligent about following up on doctor's appointments. (non-prescriber) |
| Uncertain | 3 | 9.4 | It’s very vague. (non-prescriber) |
| Reactions After Redacted Material Revealed (Section 2) | | | |
| Patient Discussion | 27 | 52.9 | I would just be honest and say, so I see that you have a diagnosis here that you’re presenting for treatment of schizophrenia but I don’t see that you’re currently prescribed an anti-psychotic.  Are you currently taking one?  Have you taken one in the past?  Did we possibly forget to list any current medications that you may have forgotten?  I would just if it were me, address it pretty upfront. (non-prescriber) |
| Concerned | 18 | 35.3 | Because if she's using, yeah, if she's using both then that could be potentially deadly. (prescriber) |
| Information Necessary | 6 | 11.8 | But yeah, there's a lot of information missing. I would want a more complete social, family history, hospitalization history, past medications. It’s again, we don't know what's worked, what hasn't worked and we're just kind of now starting from scratch again…if that's all that's there, it's not enough to move forward with treatment without more information. (prescriber) |
| Reactions to Patient Rationale for Decisions (Section 3) | | | |
| Need to Know | 33 | 68.8 | As the therapists doing an assessment, we’re not necessarily going to even get to a question that hits on all of the panels that are missing. (non-prescriber) |
| Surprised | 9 | 18.8 | I'm surprised how much is already there. Because typically they're very paranoid and very suspicious and they completely opt out of sharing any information with anyone until they're on medication. I would certainly want access to the controlled substance monitoring program and that's the best, fastest way we can look at it…Typically, people taking opiates aren't forthcoming that they're taking opiates. (prescriber) |
| Not Surprised | 6 | 12.5 | I mean, I don’t know.  It’s just maybe it is judgment and stuff but I did expect that there would be some kind of controlled substance or something on there. |
|  |  |  | (non-prescriber) |
| Rationale of Patient Decision to Redact (Section 3) | | | |
| Stigma and Fears | 13 | 43.4 | I don't know the culture of this client, but culturally they might be thinking like, ‘This person thinks I'm crazy or people will think I'm crazy because I take medication so I'm just not going to say anything.’ Particularly if it's a court-ordered client, they may be sharing less because they just want to get their mandates over with and get out of services. And the more they share could keep them wrapped up in services for longer than they want. (non-prescriber; non-prescribers nod in agreement) |
| Purposeful Omission | 7 | 23.3 | Well, I’m just saying in general, if I go to the PCP, I’m going for one thing, I don’t need 50 other things added on to what I came here for. So, maybe they’re just shutting it down. And like, look, this is what I’m here for and this is what I'm giving you. (non-prescriber) |
| Patient Considered Irrelevant | 5 | 16.7 | Or is it with the one-time [suicide] attempt, it really didn't mean nothing. I didn't really want to do it, so I'm okay now. So, it's not important to me. It’s not relevant to them. |
|  |  |  | (non-prescriber) |
| Patient Does Not Know What Information is Necessary to Share | 3 | 10 | Like six months into treatment, they suddenly randomly talk about a shopping addiction or something like that that they just never mentioned. And so, I'm sure there's some things that they don't realize are important to share [with us]. (non-prescriber) |
| Symptomatic | 2 | 6.7 | There's the possibility that they're not 100% compliant with their medication because again, there's a lot of side effects from medications. And I'm not seeing side effects of medication being prescribed and then there's the drug screen, so we don't know how much the person's self-medicating and taking their meds. So, they may be more symptomatic hence could be more paranoid about sharing the information. So, I'd want to rule that out as well. How symptomatic are they at that particular moment, you know? (non-prescriber) |
| Perceptions Toward Patient Data Categorization and Sharing (Section 5) | | | |
| Does Not Understand | 41 | 66.1 | Yeah, I mean suicidal attempt, I don't know why you would put that out there under other, compared to mental health. So, it's either stigma or education or it doesn't seem like the right category considering. (non-prescriber) |
| Patient Incorrect | 15 | 24.2 | I think it’s either the patient is purposely omitting information or they’re not educated well enough to know what each category correctly means and how to classify each thing into the correct category. I don’t know. (non-prescriber) |
| Patient Correct | 6 | 9.7 | You know, if we’re seeing [an item] as one thing that they're seen as another, this could really cause some harm. But there is a basic understanding of items, so some of this stuff makes sense minus the tramadol. (Agreement throughout) (non-prescribers) |
| Discussion of Patient Data Categorizations (Section 5) | | | |
| Other Information | 25 | 32.5 | So, then they can possibly choose to not share the right information with providers. (prescriber) |
| Genetic | 17 | 22.1 | Or even depressive disorder, could be [genetic] if they believe that it runs in the family. (non-prescriber) |
| Mental Health | 14 | 18.2 | The only part I see that doesn’t make sense is the complete blood count and the free T3, and the TSH. That is – that’s a sort of across the board test. They’re not, that’s not mental health specific. (non-prescriber) |
| Drug Use | 9 | 11.7 | What stuck out to me is OxyContin because it’s not -- I mean, it is a medication but so many people link it, and that’s a lot of the times people will come in there, you know, I’m using this and I’m not abusing it. I know you’re going to think I’m abusing it, you know. So, it’s interesting that they put that under drug abuse unless maybe they really did abuse it. Or if they just assumed we would assume that, so that’s interesting. (non-prescriber) |
| Alcohol Use | 9 | 11.7 | Well, what I was thinking with amphetamine and alcohol is maybe when they use, they use them together like when I drink I also use Amphetamine like that’s, you know, one’s in upper runs it down or I party, I get high, drink, like maybe they’re thinking that those two go hand in hand. (non-prescriber) |
| Sexual and Reproductive Health | 3 | 3.9 | We run pregnancy tests...A lot of the time just because of medication purposes. (non-prescriber) |
| Recommendations for PC-GIS (Section 5) | | | |
| Trust, Understanding Promotion | 23 | 60 | Absolutely interesting because again, the client isn't sharing information about their mental health with the people who are designated to help them with their mental health. So again, if that's the theme then trying to (A) understand what is the motivation for that and (B) is there something that can be done to assist with building some trust? If that's in some way, you know, if they don't trust the system or whatever it may be or they’re symptomatic, how can we kind of overcome that barrier in order to get that client's unique needs met? (non-prescriber) |
| Other | 7 | 17.9 | I would use a similar grid like that grading, because at a glance, you could introduce something every three months, any updates. Are you still sharing with your pharmacist? Are you still sharing with your own specialty care providers, etc.? Have you mentioned that you have an upcoming appointment with PCP? And a bit something of an alert, definitely, you need to work with the team and send an email. (non-prescriber) |
| Simplified Education Material | 4 | 10.3 | Even having it written down, sometimes it might be too much for somebody who’s having schizophrenia. If I’m hearing voices, I don’t have the patience to sit down either listen or read something. I just want to get it done as soon as possible. (non-prescriber) |
| Role-Specific Information Education | 3 | 7.7 | We also take time to educate because if we have to educate them on everything, there’s thousands of topics to discuss, and we can't educate or try to educate on things that we're not competent in. So, I can't talk to them about medications. I won’t [non-prescriber] because I can't. It's not ethical, and it's not a smart decision. So, you know, if they want the education, then they have to go see their doctor or their nurse practitioner, you know? And then it's just more steps. But if they're willing to do it, that's great. But they have to be motivated to do that. (non-prescriber) |
| Examples | 2 | 5.1 | Give an example.  Because someone with schizophrenia is not going to have the patience to sit there and listen to what each definition is and where it goes. (non-prescriber; agreement non-prescribers and prescriber) |
